# Detection and Analysis of Reactive Oxygen Species (ROS): Buffer Components Are Not Bystanders

**DOI:** 10.1021/acs.analchem.4c07070

**Published:** 2025-06-03

**Authors:** Shubham Bansal, Muskan Gori, Joanna Afokai Quaye, Giovanni Gadda, Binghe Wang

**Affiliations:** Department of Chemistry and Center for Diagnostics and Therapeutics, 1373 Georgia State University, Atlanta, Georgia 30301 United States

## Abstract

Reactive oxygen species (ROS) play critical roles in pathophysiological processes. Therefore, there is widespread interest in learning ROS concentrations under various conditions. However, literature numbers in ROS concentration vary significantly, and most cannot be readily compared against each other, largely because of the lack of understanding of the effects of various factors that significantly impact the experimental outcome. In this study, we examine an overlooked factor: the chemical reactivity of commonly used organic buffer molecules toward ROS and how such reactivity affects the results interpretation. Specifically, we examined HEPES, Tris, MES, citrate, ammonium acetate, and phosphate-buffered saline (PBS) and found that most organic buffer components can rapidly consume NaOCl (the second most abundant ROS) and/or directly interact with certain ROS probes such as a boronate for H_2_O_2_ determination, leading to significant errors in experimental findings and interpretations of results. For example, 20 mM HEPES, MES, ammonium acetate, and Tris are found to consume 1 mM hypochlorite within 1 s, leading to false negative results. Additionally, these organic buffer components have been found to cause false negative results in the detection of ONOO^–^ when using a boronate-based probe. As such, these organic buffers should be avoided in the determination of ROS concentrations. We use these examples to draw attention to the profound effects of buffer components on ROS detection and examine chemistry issues in detail. We hope the findings described will lead to improved rigor in designing ROS experiments by considering factors that were previously considered as nothing but bystanders or benign.

## Graphical abstract

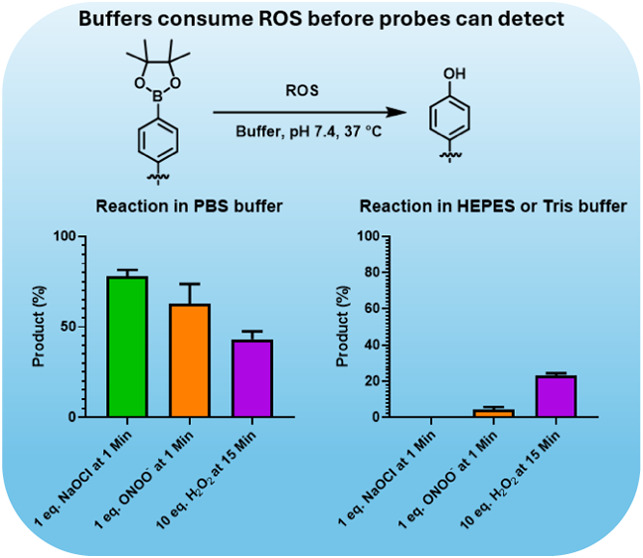

## Introduction

Reactive oxygen species (ROS) play critical roles in pathophysiological processes.^1,2^ Under normal conditions, cells maintain a redox homeostasis.^3,4^ Pathological conditions may disrupt this redox homeostasis, leading to excessive production of ROS,^5^ which in turn has been associated with conditions, such as cancer,^6–8^ viral infections,^9–12^ cardiovascular diseases,^13,14^ neurological disorders,^15,16^ chronic inflammation,^17,18^ and diabetes^19,20^ among others. Therefore, there is widespread interest in understanding ROS concentrations under various pathophysiological conditions for studying basic mechanistic questions and for ROS-sensitive delivery of drugs and/or imaging agents.^21–25^ However, literature numbers of ROS concentration vary significantly, and most cannot be readily compared against each other, largely because of the lack of understanding of the effects of various factors that significantly impact experimental outcome. A recent consensus publication in *Nature Metabolism* also highlights this issue.^26^ In earlier studies, we have shown how in-depth understanding of reaction kinetics can significantly affect results interpretations.^27,28^ Further, the use of seemingly benign organic solvents such as dimethyl sulfoxide (DMSO) for solubilizing ROS probes can have a profound impact on experimental outcomes and results interpretations.^29^

In this study, we examine a commonly overlooked factor: the chemical reactivity of commonly used organic buffer molecules toward ROS and how such reactivity affects the results interpretation. Specifically, we examined HEPES, Tris, MES, citrate, ammonium acetate, and phosphate-buffered saline (PBS) and found that most organic buffer components can rapidly consume the second most abundant ROS: NaOCl, leading to false negative results. Additionally, these organic buffers were found to cause false negatives in the detection of ONOO^–^ when using a boronate-based probe. Furthermore, organic buffers were found to directly interact with certain ROS probes, such as a boronate for H_2_O_2_ determination. Overall, these organic buffers were found to cause significant errors in experimental findings and interpretation of results.

Below, we describe our studies and findings.

## Experimental Section

### Boronate Reaction with NaOCl in Commonly Used Buffer Solutions

First, stock solutions were prepared. A 10 mM solution of 4-acetylphenylboronic pinacolate ester (APBE) **1** was prepared in dimethylformamide (DMF) by dissolving 2.884 mg in 1.171 mL of DMF. Then, 10 μL was taken from the 10 mM stock solution of APBE **1** and added to 980 μL of buffer. This was followed by the addition of 10 μL of 10 mM NaOCl solution. The reaction mixture had a final concentration of 100 μM APBE **1** and 100 μM NaOCl in 10 mM different buffer solutions containing 1% DMF at 37 °C. Then, a 20 μL aliquot was drawn from the reaction mixture and injected into high-performance liquid chromatography (HPLC). Mobile phase gradient method A was used to monitor the product formation.

### Reaction of Boronate with H_2_O_2_ in the Following Buffers: HEPES, Tris-Cl, and PBS

First, stock solutions were prepared. 10 mM stock solution of APBE **1** was prepared in DMF by dissolving 2.884 mg in 1.171 mL of DMF. Then, 1 mM working solution of APBE **1** was prepared by adding 100 μL of the stock solution of APBE **1** (10 mM) to 900 μL of DMF. Ten millimeters working solution of H_2_O_2_ was prepared in H_2_O. Then, 10 μL of APBE **1** stock solution (1 mM) was added to 980 μL of the 10 mM buffer. Then, 10 μL of a 10 mM H_2_O_2_ solution was added to the mixture. The resultant reaction mixture was incubated at 37 °C. The reaction mixture had a final concentration of 10 μM APBE **1** and 100 μM H_2_O_2_ in different buffers (10 mM) with 1% DMF at pH 7.4. At designated time points, 20 μL aliquots were drawn from the reaction mixture and injected into HPLC. Gradient method B was used to monitor the product formation. All the buffer solutions were at pH 7.4.

## Results and Discussion

ROS studies are important for understanding its pathophysiological roles and signaling functions, ROS-sensitive drug delivery, and imaging work. Regardless of the ultimate goal, there is usually involves a reaction-based probe in such studies.^30^ Among all of the ROS, the most widely studied are H_2_O_2_ and hypochlorite because of their relatively high abundance and long-lived nature.^27^ H_2_O_2_ concentration in various pathological diseases has been reported to be as high as 610 μM, making it “the most abundant species.”^27^ Whereas H_2_O_2_ concentration in normal physiological conditions has been reported as in the single-digit micromolar range.^27^ For example, human plasma H_2_O_2_ concentration has been reported to be 3 μM.^31^ Naturally, this over 200-fold difference in the H_2_O_2_ level can be exploited for triggered prodrug activation or imaging work. For hypochlorite, the situation is similar. For example, hypochlorite has been reported to be present in the concentration range of 20–85 μM in unstimulated cancer cells such as HepG2,^32^ MCF-7,^32^ LO2,^32,33^ 298T,^33^ and HT-29.^32^ In animal model studies, hypochlorite concentrations have been reported to be as high as 100 μM in injured liver in mice induced by alcohol and ∼422 μM in injury induced by acetaminophen.^27,33^ In neutrophils, NaOCl concentration has been reported as high as 398 μM upon stimulation.^34^ Nevertheless, because of differences in the method used in each determination, these numbers cannot always be readily compared.^27^ Further, most of these concentrations were determined by using reaction-based probes by observing production formation over a defined period of time. Therefore, these concentrations are essentially “cumulative” concentrations. The actual value depends on the probe chemistry used, reaction kinetics, duration of the experiments, whether the sample represents a snapshot or in live cell/tissue with the ability to continuously produce ROS, and interference by other ROS. For example, in cancer cells, H_2_O_2_ concentration has been reported in the range of 0.8–610 μM.^27^ Such a wide range may have pathophysiological reasons or variations in experimental methods. We have recently examined some of these issues.^27^ All of these indicate the need to carefully examine the chemistry issues in ROS research in order to generate reliable ROS concentration data for individual species that can be compared with each other. Such information is the foundation for understanding ROS biology at the molecular level.

In our ROS-related research, we have observed what could be considered idiosyncratic variations of experimental results depending on changes in seemingly benign factors such as the organic solvent used for solubilization and buffer. However, such an idiosyncrasy means that there were factors that we did not understand. For an in-depth understanding of factors that influence such experiments, we have recently described the effect of an organic solvent such as DMSO on experimental findings.^29^ In this study, we examine the significant effects of organic buffer molecules on the identification and concentration determination of ROS. Specifically, HEPES, Tris, MES, ammonium acetate, and citrate are commonly used buffer components in ROS-related studies. For example, we surveyed 50 publications related to hypochlorite, peroxynitrite, and H_2_O_2_ research and found the use of at least one of these buffer components in about 60% of the publications. The chemical structures of these buffer components indicate a strong propensity for these molecules to react with certain ROS and to interact with some probes used for such determinations (Figure 1). Therefore, we became interested in examining the effects of buffer components on the interactions among representative probes with H_2_O_2_, ONOO^–^, and NaOCl, respectively. We intend to highlight issues to consider in designing future ROS experiments, not to be comprehensive because of the vast number of possibilities. Below, we describe how these buffers have a profound impact on experimental outcomes when dealing with ROS.

**Figure 1:**
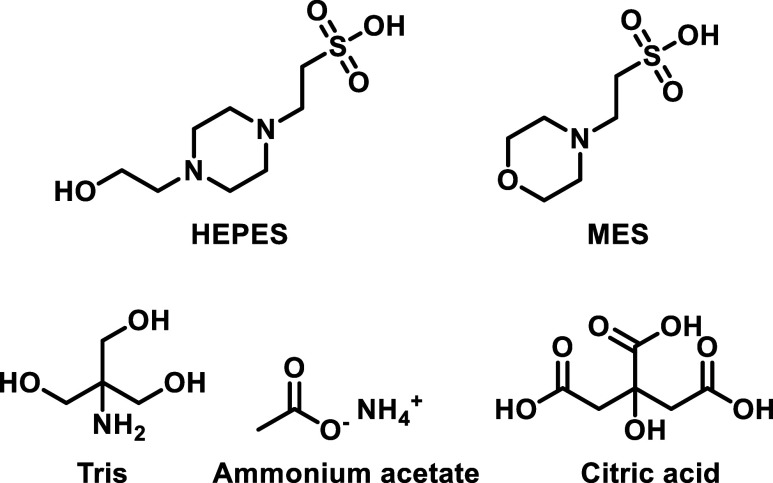
Structures of the commonly used buffer components.

### Buffer Reactivity with NaOCl

As the first step, we studied the reactivities of NaOCl with different buffer components. NaOCl has a λ_max_ of 292 nm with a molar extinction coefficient of 360 M^–1^ cm^–1^,^35^ allowing for its monitoring at mM concentrations by ultraviolet–visible (UV–vis) spectrophotometry. Specifically, we used buffer components at 20 mM, which is at the lower end of the buffer concentrations commonly used. We started by studying the reaction of NaOCl (2 mM) at pH 7.4 and 37 °C with HEPES, which has no meaningful absorption in the region 250–400 nm. Upon incubation for 1 min, the peak at 292 nm disappeared to an undetectable level with concomitant appearance of a peak at 260 nm, indicating consumption of NaOCl by HEPES and the formation of a new chromophoric compound (Figure 2a). Looking at the structure of HEPES, one can readily see several oxidizable functional groups, including amino and hydroxyl groups. NaOCl is known to readily oxidize an amino group to chloramine.^36^ Incidentally, chloramine is known to have a λ_max_ close to 260 nm (λ_max_ of 244 nm for the product of ammonia and NaOCl).^35^ At the 5- and 30 min time points, the intensity at 260 nm also further decreased, presumably because of degradation or further reactions by chloramine. Indeed, chloramines are known to have stability issues.^37–40^ HEPES has tertiary amines, which can form quaternary chloramine ions after reaction with NaOCl.^41^ Such quaternary chloramine species can rapidly degrade, leading to a decrease in chloramine concentration.^40,41^ Without the need to study the detailed chemistry, one outcome is unambiguous: there is strong interference from HEPES in hypochlorite-related studies.

**Figure 2:**
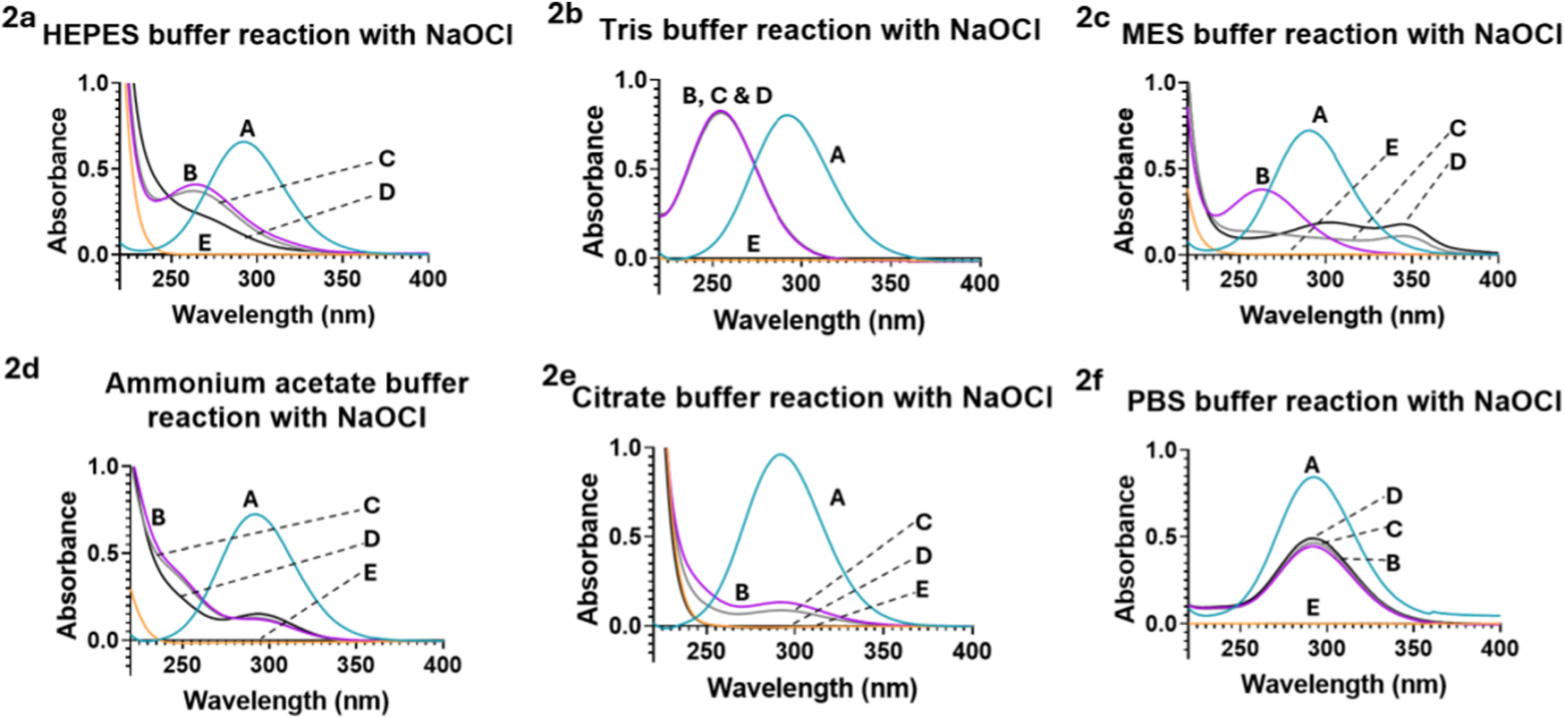
Reactions of commonly used buffers with hypochlorite monitored by UV–vis spectrophotometer. (2a–f) Reaction of NaOCl with buffer molecules at 37 °C; (2a): HEPES at pH 7.4; (2b): Tris at pH 7.4; (2c): MES at pH 6.3; (2d): Ammonium acetate molecules at pH 4.5; (2e): Citrate at pH 5.9; (2f): PBS at pH 7.4. (2a-f) **A**: Spectrum of 2 mM NaOCl in H_2_O (Standard); **B**: Spectrum of the reaction mixture of 2 mM NaOCl and 20 mM respective buffer component at 1 min. **C**: 5 min. **D**: 30 min. **E**: Spectrum of 20 mM of the respective buffer component (Standard).

After observing such a fast consumption of NaOCl by HEPES, we next studied the same with Tris-Cl, MES, ammonium acetate, citrate, and PBS. Indeed, NaOCl showed spectral changes upon the addition of each of the buffer compounds tested. Starting with Tris-Cl, the reaction seems to be very fast with the total disappearance of the peak at 292 nm (NaOCl) at the 1 min point and concomitant appearance of a peak at 250 nm, presumably corresponding to the chloramine product (Figure 2b).^35^ Tris contains a primary amino and three hydroxyl groups, both of which are known to react with NaOCl, leading to the formation of chloramines and alkyl hypochlorite, respectively.^36,42^ The spectrum of the Tris and NaOCl reaction mixture did not show any change within from 1 to 30 min (Figure 2b). This is different from that of the HEPES reaction and indicates a higher level of stability for the chloramine product from Tris. Such a difference in degradation propensity is consistent with literature stability reports of chloramines of primary and tertiary amines.^40,43^

As expected, MES, ammonium acetate, and citrate buffers all showed reactivity with NaOCl (Figure 2). All three buffers (MES, ammonium acetate, and citrate) showed NaOCl consumption within 1 min. In reactions with MES and ammonium acetate, a new peak at around 250 nm was observed, indicating the formation of the chloramine product. Further spectral changes were observed at the 5 and 30 min points, indicating degradation for the chloramine formed. Similar to HEPES, MES also has a tertiary amine, which can form quaternary chloramine ions after reaction with NaOCl^41^ and rapidly degrade, leading to decreased concentration of the chloramine.^40,41^ Looking at the structure of citrate, one would not expect to see chloramine formation because it lacks an amino group. Therefore, in the citrate and NaOCl reactions, we only observed a decrease in intensity at 292 nm without the concomitant formation of a new peak. The reaction of NaOCl with citrate has been reported to lead to the formation of alkyl hypochlorite,^42,44,45^ which is still very reactive.^42^ Further, the reactivity of alkyl hypochlorite can be different from that of NaOCl.^42,46–48^ We emphasize that we do not intend to study the detailed reaction mechanism(s) and products from these buffer components. We are focused on showing the fact of buffer interference and raising a cautionary note so that others can pay particular attention to this issue when conducting their own ROS-related experiments, when applicable.

It should be noted that incubation of NaOCl with PBS also led to a decrease of the peak at 292 nm by about 50% at the 1 min time point, with no further changes within the period of 1–30 min (Figure 2f). The UV absorption of NaOCl is known to be pH-dependent.^35^ Hypochlorous acid of 2.13 mM has been reported to have an absorbance of about 0.8 at pH 12 and about 0.4 at pH 7.4.^35^ Dissolution of the commercial sodium NaOCl led to a solution at pH 12; whereas PBS was kept at pH 7.4. Therefore, the absorbance drop of 50% after dissolving in PBS at 7.4 is consistent with the known pH effects.^35^ Later experiments in the section on “**Boronate reaction with NaOCl in commonly used buffers**” also confirmed that NaOCl is stable in PBS (Figures 4 and S11). Next, we were interested in determining the reaction rate constant in order to understand issues related to possible interference from buffer molecules by taking into consideration kinetic factors.

### NaOCl Reaction Kinetics with Commonly Used Buffer Components

With the findings of strong reactivity of NaOCl with the various buffer compounds, next we were interested in understanding the reaction kinetics to put the issue of possible interferences by various buffer components into a proper context. Because of the fast reaction of NaOCl with buffer components, we decided to use stopped-flow spectrophotometry for subsequent reaction rate studies. Briefly, the reaction of 1 mM NaOCl and 20 mM each buffer compound was studied (Figure S1) by monitoring absorption changes at 325 nm, which was determined based on the observed signal-to-noise ratio. At 20 mM HEPES and 1 mM NaOCl, complete NaOCl consumption was observed in about 0.1 s (Figure 3a). The pseudo-first-order rate constant was determined to be 177 s^–1^ by a single exponential decay (Figure S2 and Table 1). The kinetics traces showed an initial plateau phase of about 1.73 ms. Though the reaction mechanism was not the focus of this study, we did consider various possibilities for the initial lag period. Since reaction progression was monitored by measuring the disappearance of hypochlorite at 325 nm, the lag period could mean the formation of a very reactive intermediate species with the same UV characteristics. The most likely species is chloramine of HEPES. However, chloramine is known to have a UV λ_max_ at about 244 nm.^35^ Further, chloramine is much less reactive than hypochlorite.^38^ If one uses the reaction of an amine with chloramine or NaOCl as a gauge, the reactivity difference is on the order of 10^9^ with the second-order rate constant being 0.32 M^–1^ s^–1^ for the reaction with chloramine^38^ and 10^8^ M^–1^ s^–1^ for the reaction with hypochlorite.^49^ Therefore, chloramine is unlikely to be the contributing species. We did not further pursue other factors, because that would be beyond the scope of this study. The finding of the rapid and complete consumption of NaOCl within 100 ms by HEPES clearly indicates the need to avoid this buffer when studying hypochlorite concentrations.

**Table 1:** Pseudo-First-Order Rate Constants and Half-Life Calculations of NaOCl Reaction with Commonly Used Buffer Components^a^

#	buffer (20 mM)	pseudo-first-order rate constant (s^–1^)	NaOCl consumes with *t*_1/2_
1	HEPES	177	3.9 ms
2	MES	14	49 ms
3	Tris-Cl	2092^b^	0.3 ms
4	CH_3_COONH_4_	2092^b^	0.3 ms

a Reaction rates are based on the reaction of 1 mM NaOCl and 20 mM buffer component.

b Reaction completed in the instrument mixing time of 2.2 ms, so the lower limit of the rate constant is theoretically calculated based on the instrument mixing time.

**Figure 3:**
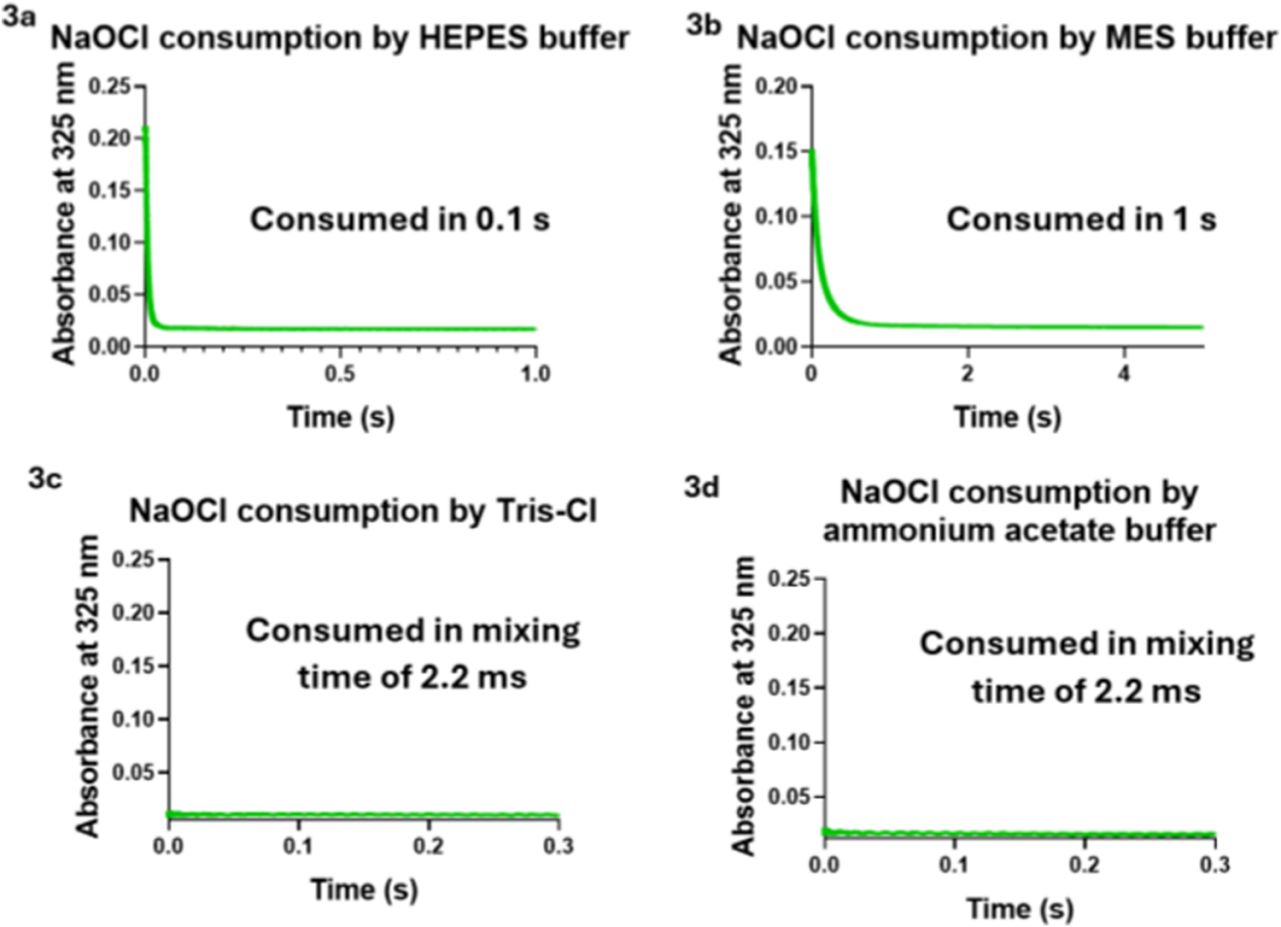
NaOCl consumption by commonly used buffers monitored by stopped flow. (3a) Reaction of 1 mM NaOCl and 20 mM HEPES at pH 7.4 and 37 °C. (3b) Reaction of 1 mM NaOCl and 20 mM MES at pH 6.3 and 37 °C. (3c) Reaction of 1 mM NaOCl and 20 mM Tris-Cl at pH 7.4 and 37 °C. (3d) Reaction of 1 mM NaOCl and 20 mM ammonium acetate buffer at pH 4.5 and 37 °C.

In an attempt to determine the second-order rate constant, we first measured the pseudo-first-order rate constants by using HEPES in excess: 1 mM NaOCl with HEPES at 25, 30, 35, 40, 45, 50, and 60 mM, respectively (Figure S3). The plan was to plot the pseudo-first-order rate constants against HEPES concentrations to determine the second-order rate constants. Much to our surprise, the pseudo-first-order rate constant decreased with increasing HEPES concentration (Figures S3 and S4), contrary to our expectations. In our years of conducting similar kinetic experiments, we have never encountered this problem. One possible reason for this decreased pseudo-first order rate constant with increasing HEPES concentration could be due to self-association, which could change the reactivity of the nitrogen atom toward NaOCl. The piperazine moiety present in HEPES is known to be prone to self-association, though there is no report of self-association of HEPES.^50^ This self-association could be the reason for the decreased pseudo-first-order rate constant with elevating HEPES concentration (Figure S4). Regardless of the specific rate constant, HEPES consumes NaOCl in less than 1 s at all HEPES concentrations (Figure S3). Additionally, a literature value determined using an indirect method also indicates the rapid consumption of NaOCl by HEPES (4400 M^–1^ s^–1^).^51^ Without the need to study further details, one outcome is unambiguous: there is strong interference from the HEPES in NaOCl-related studies.

MES showed a slower reaction rate compared to HEPES but still consumed all of the NaOCl in about 1 s under the same conditions (Figure 3b). The kinetic traces for MES seem to show two phases, probably indicating secondary reaction(s). As a result, the pseudo-first-order rate constant was determined as 14 s^–1^ for the first phase by using a double-exponential method of 20 mM MES and 1 mM NaOCl (Figure S5 and Table 1). For the second phase, tertiary amines are known to form quaternary chloramine ions after reaction with NaOCl.^41^ Such a quaternary chloramine species can undergo degradation, leading to the formation of an aldehyde and a secondary amine after hydrolysis.^40,41^ The secondary amine formed in this reaction can also react with NaOCl; this could be a reason for the second phase when the NaOCl concentration changes. As discussed before, we did not further pursue the issues of degradation products, as that would be beyond the scope of this study. Regardless, MES consumed all of the NaOCl within 1 s (Figures 3 and S6). We determined the pseudo-first-order rate constants by using MES in excess: 1 mM NaOCl with MES at 25, 30, 35, 40, 45, 50, and 60 mM, respectively (Figure S6). Then, the pseudo-first-order rate constant was plotted against MES concentration to yield the second-order rate constant of 958 ± 30 M^–1^ s^–1^ based on the slope (Figure S7).

For the reactions between NaOCl and Tris-Cl or ammonium acetate, respectively, the reaction was found to be complete, even before the first spectral scan by the stopped-flow instrument (Figure S1). The observation suggests that the reaction occurred within the mixing time of the stopped-flow instrument, which is about 2.2 ms (Figure 3), giving a pseudo-first-order rate constant of 2092 s^–1^ as the lower limit. This provides the lower end of the second-order reaction rate being about 1.0 × 10^5^ M^–1^ s^–1^. For comparison, the reported second-order rate constant for reactions between a methyl amine and NaOCl is about 1.9–3.6 × 10^8^ M^–1^ s^–1^.^49^ Regardless of the specific number for the rate constant, all of the indications are that the reaction between NaOCl and Tris-Cl or ammonium acetate is very fast. For normal ROS-related experiments on time scales of min to h, there is no meaningful difference when the reactions between the buffer component and NaOCl are this fast because these buffer components can consume NaOCl within a few seconds.

The reaction kinetic information will inform the degree of interference, depending on the reaction in question. For example, the second-order rate constant of methionine oxidation by NaOCl is 3.7 × 10^8^ M^–1^ s^–1^,^52,53^ which is significantly (5 orders of magnitude) faster than the oxidation of HEPES. Then, one would not expect to see meaningful competitive reaction of HEPES with NaOCl unless HEPES is in excess by more than 5 orders of magnitude. On the other hand, boronate oxidation by NaOCl has a second-order rate constant 5.7 × 10^3^ M^–1^ s^–1^,^54^ which is similar to the second-order rate constant of HEPES with NaOCl (4.4 × 10^3^ M^–1^ s^–1^).^51^ Then, one would expect significant interference of boronate oxidation by HEPES because HEPES and boronate have comparable reactivity with NaOCl and yet HEPES is present in excess. Naturally, for a probe having a reaction rate with NaOCl that is slower than that with HEPES, one would expect severe interference by this buffer component.

Overall, all of the organic buffer components studied showed a very fast reaction with NaOCl, leading to consumption of NaOCl within seconds (Figure 3 and Table 1). The pseudo-first-order rate constants under the experimental conditions and the half-life of NaOCl consumption by these buffers are summarized in Table 1.

### Effect of Buffer on the Reaction Between a Probe and ROS

Though the previous section described the consumption of NaOCl by various organic buffer components, it is recognized that the products from such reactions (i.e., chloramine and alkyl hypochlorite) are still very reactive. Then, there is the question of whether the commonly used probes can still detect these secondary products at an efficiency similar to that of NaOCl. We next examined whether or not the presence of these buffer components interferes with the ability to determine NaOCl concentration using commonly used probes. For this, we used two examples. The first molecule is a boronate-containing model compound, which represents a large number of fluorescent probes for ROS studies.^25,55–61^ The second probe is 2,7-dichlorodihydrofluorescein (DCFH), which is commonly used to determine ROS concentration.^62–64^

### Boronate Reaction with NaOCl in Commonly Used Buffers

The oxidation of a boronic acid moiety by peroxide is a commonly used approach for designing fluorescent probes for various ROS. For example, Chang and colleagues developed a number of boronate-based probes for the detection of ROS.^25^ Such a reaction involves a peroxy anion attacking the boron atom with an open shell, followed by rearrangements leading to the formation of a hydroxyl group in the position of the boronic acid moiety (Scheme 1). Because of the widespread use of such boronic acid chemistry, we started by examining the effect of buffer components on the reaction of a boronate with NaOCl. We chose 4-acetylphenylboronic pinacolate ester (APBE) **1** as a model compound for this study. This selection was made because of its known rate constant 5.7 × 10^3^ M^–1^ s^–1^ in its reaction with NaOCl and its simplicity.^54^ Briefly, we examined the effect by adding a stoichiometric amount of NaOCl to a solution of APBE **1** (100 μM) in different buffers and analyzed the products using HPLC. We first conducted a control reaction by incubating APBE **1** with NaOCl in PBS without DMSO and observed the quick and quantitative conversion of APBE **1** to a new peak corresponding to the oxidized product **2** (Figure 4). Such results also help confirm that the decreased UV absorbance of NaOCl in PBS (relative to NaOCl alone; Figure 2f) was due to pH changes, not NaOCl consumption. Interestingly, when the same reaction was conducted in HEPES, MES, Tris-Cl, or ammonium acetate buffer by adding NaOCl to a solution of APBE **1** in the respective buffer, no product **2** formation was observed (Figure 4). Obviously, HEPES, MES, Tris-Cl, and ammonium acetate were able to react with NaOCl fast enough to prevent APBE **1** oxidation. Furthermore, the chloramine products from such reactions do not react with boronate within the time scale studied. Because HPLC was used to study reaction profiles, there is the question as to whether HPLC mobile phase (H_2_O, ACN, and trifluoroacetic acid (TFA)) might interfere with the outcome (Figure 4) due to the NaOCl reaction with mobile phase. Therefore, we conducted experiments with ACN and TFA and were able to rule out the interference from ACN or TFA during HPLC analysis. First, NaOCl consumption was complete within 1 s when Tris, HEPES, MES, or ammonium acetate was used (Figure 3). Therefore, by the time of HPLC injection, no NaOCl is expected to remain to interfere with reactions. The results in Figure 4c indicate so. For the reaction in PBS, a second-order rate constant is 5.7 × 10^3^ M^–1^ s^–1^ for the reaction of boronate with NaOCl,^54^ giving a first calculated *t*_1/2_ of 1.7 s at 100 μM each. The reaction kinetics indicate that 95% of the reaction will be completed in less than 60 s, which should lead to an almost complete consumption of APBE **1** by the time of the HPLC injection. Indeed, the reaction in PBS buffer showed complete conversion of APBE **1** to the product (Figure 4). Third, we incubated ACN with hypochlorite and saw no consumption of NaOCl (Figure S8). Fourth, we observed the lack of impact on APBE **1** integrity by a TFA-hypochlorite combination (Figure S9). We should note that the reaction kinetics of NaOCl are similar for APBE **1** and HEPES with the second-order rate constant being 5.7 × 10^3^ M^–1^ s^–1^ and 4.4 × 10^3^ M^–1^ s^–1^, respectively.^51,54^ Incidentally, both are faster than many click reactions.^65,66^ Presumably, because HEPES is present in large excess of the probe (boronate), the majority of the NaOCl was consumed by the buffer component, even if NaOCl was added after APBE **1** addition, leading to skewed results for ROS concentration determination.

**Figure 4:**
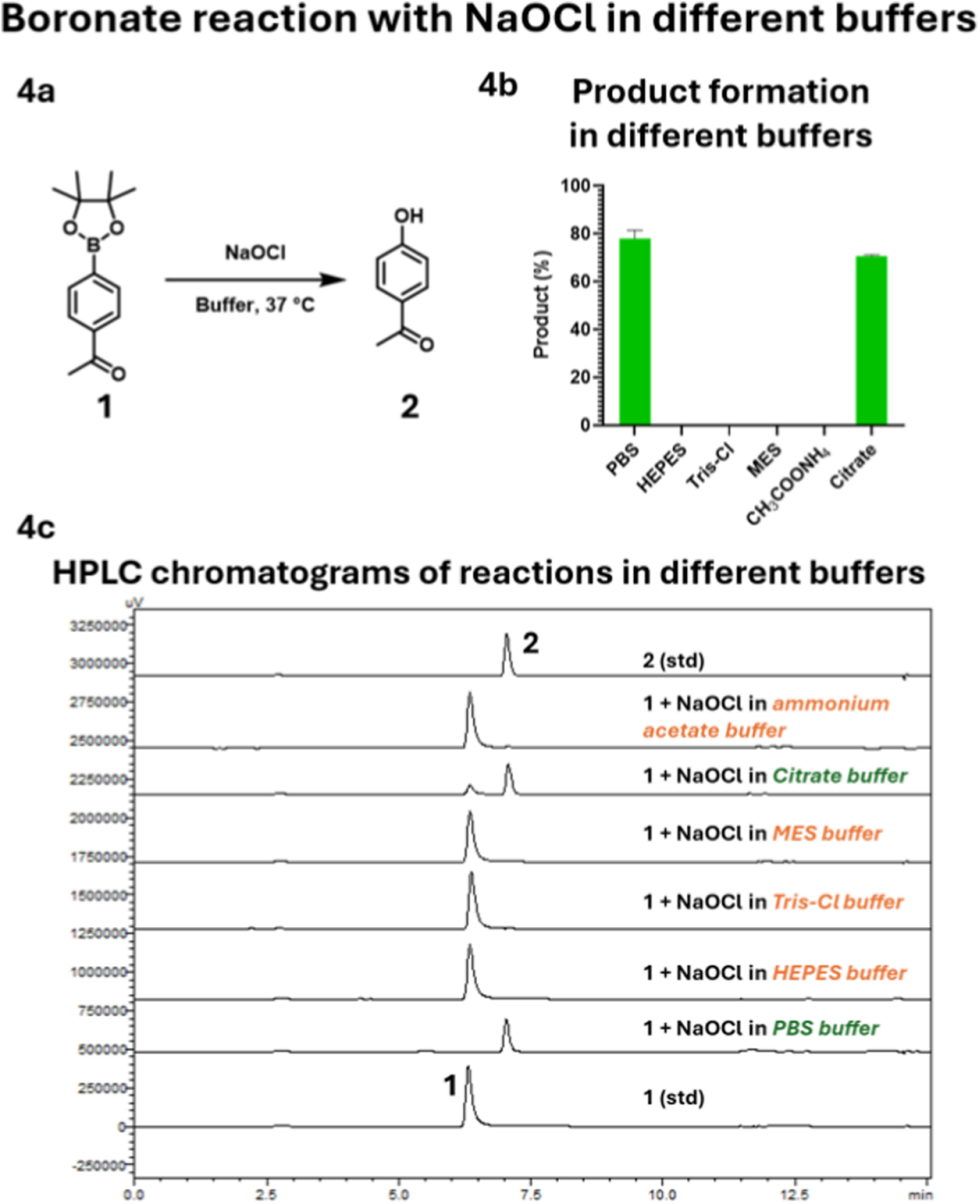
(4a) General reaction scheme of the NaOCl reaction with APBE **1** in 10 mM buffer at 37 °C. (4b) Product formation in each buffer. (4c) HPLC chromatograms showing the reaction progress in each buffer. The reagents are added in this order as first, APBE **1** was added to the buffer, and then NaOCl was added to it.

**Scheme 1:**
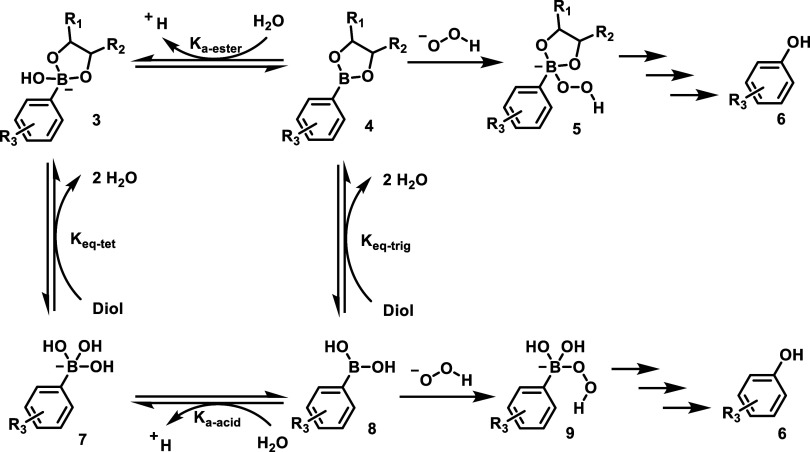
Boronate Chemistry: The Relationship between Phenylboronic Acid and its Diol Ester and Its Effects on Oxidation Reaction

It is interesting that when citrate buffer was used following the same procedures, we observed the same results as those in PBS (Figure 4). This was initially intriguing because citrate consumed the majority of the NaOCl within 1 min (Figure 2e). To gain further insights into this mechanistic question, we studied the effect of the citrate buffer by reversing the order of reagent addition. Briefly, we incubated the solution of NaOCl and citrate for 15 min at 37 °C before APBE **1** addition. We know from previous experiments (Figure 2e), 15 min of preincubation should lead to substantial, if not total, consumption of NaOCl before APBE addition. However, HPLC analyses showed product (**2**) formation in citrate buffer irrespective of the order of the reagent addition (Figure S10). It is known that reaction of NaOCl with citrate leads to the formation of alkyl hypochlorite,^42,44,45^ which is still a very reactive species.^42^ The results demonstrate that both hypochlorite and alkyl hypochlorite are reactive enough with a boronic acid compound, leading to its oxidative deborylation. Again, chloramines formed from HEPES, MES, Tris-Cl, and ammonium acetate did not react with APBE **1**. We also conducted a control experiment in PBS by reversing the order of the reagent addition. Briefly, we first added NaOCl to the PBS solution. This was followed by incubation for 15 min at 37 °C and then addition of APBE **1**. The HPLC results showed product (**2)** formation in PBS regardless of the order of the reagent addition (Figure S11). Overall, the results demonstrated significant interference depending on which buffer is used for determining the reactivity of the probe with ROS. Additionally, these results indicate that there is a need to incorporate positive control to validate the protocol when dealing with each ROS. After observing such drastic changes in the results using NaOCl, we extended the same study to ONOO^–^ and H_2_O_2_ detection.

### Reaction of Boronate with ONOO^–^ in Commonly Used Buffers

Boronate-based probes are also being used for the detection of ONOO^–^,^60,61,67^ because of the rapid reaction of boronate with ONOO^–^.^54^ The second-order rate constant of boronate reaction with ONOO^–^ reaction has been reported as 1.2 × 10^6^ M^–1^ s^–1^.^54^ However, there are the following additional complexities we needed to consider while working with ONOO^–^. First, the ONOO^–^ is known to show pH-dependent decomposition.^68^ Specifically, at pH 7.4 the ONOO^–^ has a *t*_1/2_ of 1.9 s.^68^ Second, ONOOH and ONOO^–^ have significantly different reaction kinetics.^52,69^ For example, the second-order rate constants of the reaction of ONOOH and ONOO^–^ with methionine have been reported to be 2 × 10^3^ M^–1^ s^–1^ and 2 × 10^–1^ M^–1^ s^–1^, respectively.^52,69^ Third, boronate reacts 10^3^-fold faster with ONOO^–^ compared to NaOCl.^54^ To analyze the interference from buffer molecules in ONOO^–^ detection without the competitive reaction of boronate with ONOO^–^, we first added the ONOO^–^ to the buffer solution followed by a stoichiometric amount of APBE **1** (100 μM). The reaction was analyzed using HPLC. As expected, quantitative product formation was observed only in PBS, whereas in other organic buffers, no significant product formation was observed (Figure 5). The quantitative product formation in PBS and the lack of significant product formation in HEPES and Tris indicate the ONOO^–^ consumption by the buffer molecules. These results are consistent with the literature reports of the ONOO^–^ reactivity with the functional groups present in these organic buffers.^70–72^ Overall, the results demonstrate significant interference from organic buffers in the ROS concentration determination.

**Figure 5:**
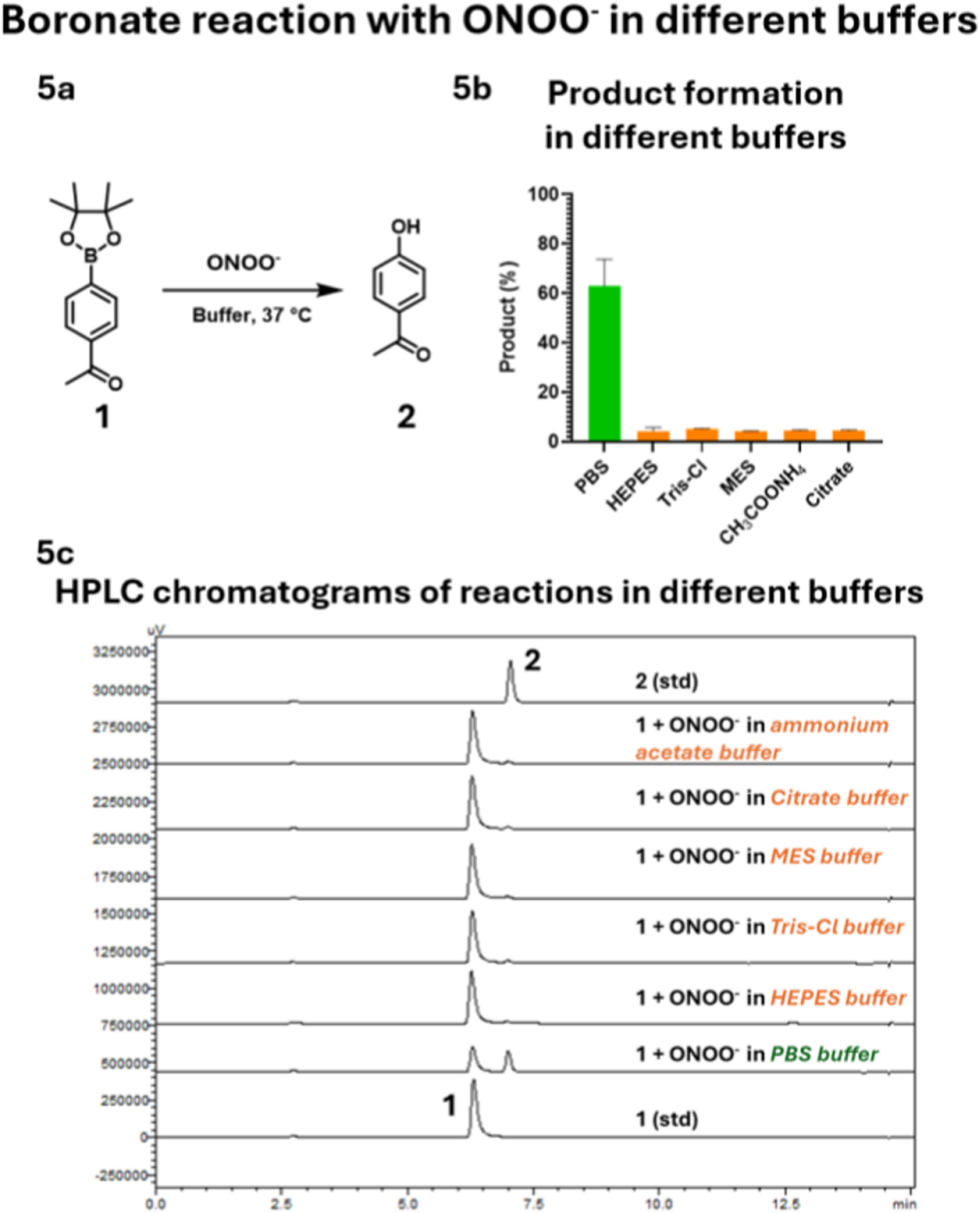
(5a) General reaction scheme of the ONOO_–_ reaction with APBE **1** in 10 mM buffer at 37 °C. (5b) Product formation in each buffer (5c) HPLC chromatograms showing the reaction progress in each buffer. The reagents are added in this order as first, ONOO^–^ was added to the buffer, and then APBE **1** was added to it.

### Reaction of Boronate with H_2_O_2_

Because boronate-based probes are also commonly used for detecting H_2_O_2_,^25,55–58^ we are interested in examining buffer effects in such studies. Though they are commonly referred as being selective for H_2_O_2_, boronate compounds react faster with other ROS such as NaOCl and ONOOH than with H_2_O_2_, by more than 2000-fold and a million-fold, respectively.^54^ To study the effect of the buffer component on the boronate reaction with H_2_O_2_, we chose three buffer solutions, PBS, HEPES, and Tris-Cl at the same pH for ease of comparison. APBE **1** has a second-order rate constant of 2.2 M^–1^ s^–1^ for its reaction with H_2_O_2_, giving a first *t*_1/2_ of 13 h at 10 μM each.^54^ Because of this slow reaction kinetics, we studied the reaction with a 10-fold excess of H_2_O_2_. Briefly, we incubated 10 μM of APBE **1** with 100 μM of H_2_O_2_ in different buffer solutions at 37 °C. Then, 20 μL aliquots were sampled every 15 min for HPLC analyses. As expected, the rates of product formation in each buffer were different (Figure 6). The reaction carried out in PBS reached near completion in 2 h (Figure 6), whereas in HEPES or Tris-Cl, the reaction did not complete in 2 h (Figure 6). At the 15 min time point, almost 40% of product **2** was formed in PBS and only ∼22% was formed in HEPES or Tris (Figure 6). At the 2 h time point, almost 100% of product **2** formed in PBS and only about 73 and 66% of product formed in HEPES and Tris-Cl buffer, respectively (Figure 6). The results may seem surprising since H_2_O_2_ is not known to be a strong enough oxidizing agent to oxidize HEPES or Tris within the time frame of the experiments.^73^ The rate constants for reactions between a tertiary amine and H_2_O_2_ have been reported to be on the scale of 10^–5^ M^–1^ s^–1^, too slow to be an interference of the reaction between a boronate and H_2_O_2_.^74^ Then, what could be the reason for the observed interference by such buffer components? In order to explain the buffer effect, it is important to recognize the Lewis acid nature of the boron atom. With its open shell, boron is prone to reaction with an electron-rich species, which is an essential step in oxidation by a peroxide (Scheme 1). In the case of APBE, pinacol ester **4** at low mM concentration is known to quickly reverse (hydrolyze) to its boronic acid **8** form in aqueous solution (Scheme 1).^75^ This free boronic acid **8** species has an open shell and is prone to oxidative cleavage (Scheme 1). In the case of APBE **1** at low μM concentrations as in the experiments conducted, hydrolysis is expected to be rapid. Because the p*K*_a_ of 4-acylboronic acid **8** is expected to be at least 7.8,^76^ it exists mostly in the oxidation-prone free boronic acid **8** form at pH 7.4.^77–79^ However, boronic acid **8** is known to have fairly high affinities for polyols and amino alcohols, including diethanolamine^80^ and sugars.^77,78^ It is conceivable that the structural properties of HEPES and Tris afford them the ability to chelate to boronic acid and keep a portion in the anionic tetrahedral form, which is not prone to oxidative degradation. All of these could be the reason for the observed interference by HEPES and Tris. Overall, these results indicate slowed product formation from H_2_O_2_-mediated oxidation of boronate in HEPES and Tris-Cl buffers than in PBS.

**Figure 6:**
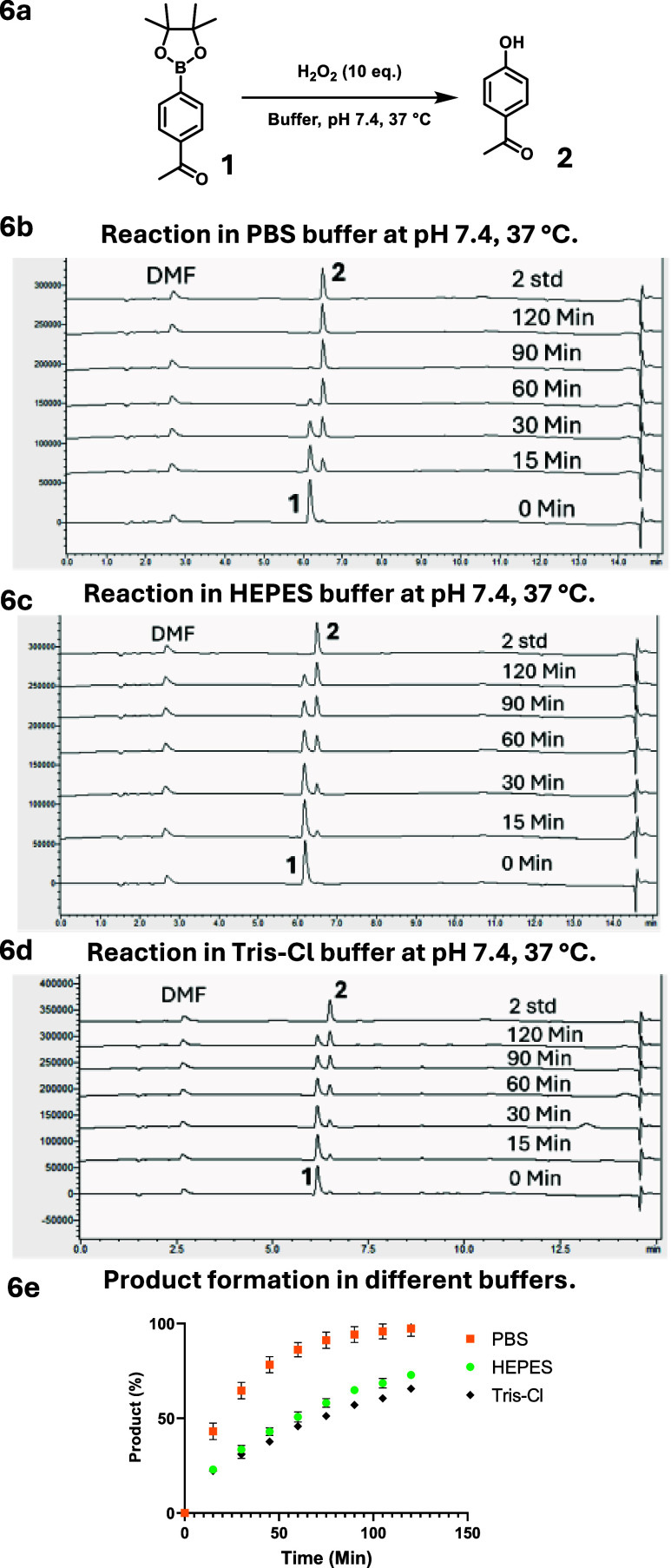
Effect of buffer on the boronate reaction with H_2_O_2_. (6a) General reaction scheme of H_2_O_2_ reaction with APBE **1** in 10 mM buffers at pH 7.4 and 37 °C. (6b, d) HPLC chromatograms showing the reaction progress in 10 mM buffer under near physiological conditions. PBS; 6c: HEPES; 6d: Tris-Cl. (6e) Product formation in each buffer with time.

### Effect of Buffer Reactivity on Fluorescent Probe, DCFH

Beyond boronate-based probes, we also examined 2,7-dichlorodihydrofluorescein (DCFH), a commonly used fluorophore for studying ROS.^62–64^

### DCFH Oxidation Kinetics with NaOCl

DCFH is known to show rapid response toward NaOCl.^29^ We started by determining the second-order rate constant of the DCFH reaction with NaOCl, using stopped-flow spectrophotometry. Briefly, we determined the pseudo-first-order rate constant by using NaOCl in excess. Specifically, we first determined the pseudo-first-order rate constant of the reaction between 20 μM DCFH and NaOCl at 250, 300, 350, 400, 450, and 500 μM (Figure S12). All of the solutions were prepared in 10 mM PBS at pH 7.4. Then, the pseudo-first-order rate constants were plotted against NaOCl concentration (Figures 7, S12, and S13), yielding a second-order rate constant of 580 ± 18 M^–1^ s^–1^ (Figures 7, S12, and S13). Such a number is smaller than that for the same reaction of NaOCl with HEPES, MES, Tris, and ammonium acetate. Therefore, we anticipate interference issues using these buffer molecules.

**Figure 7:**
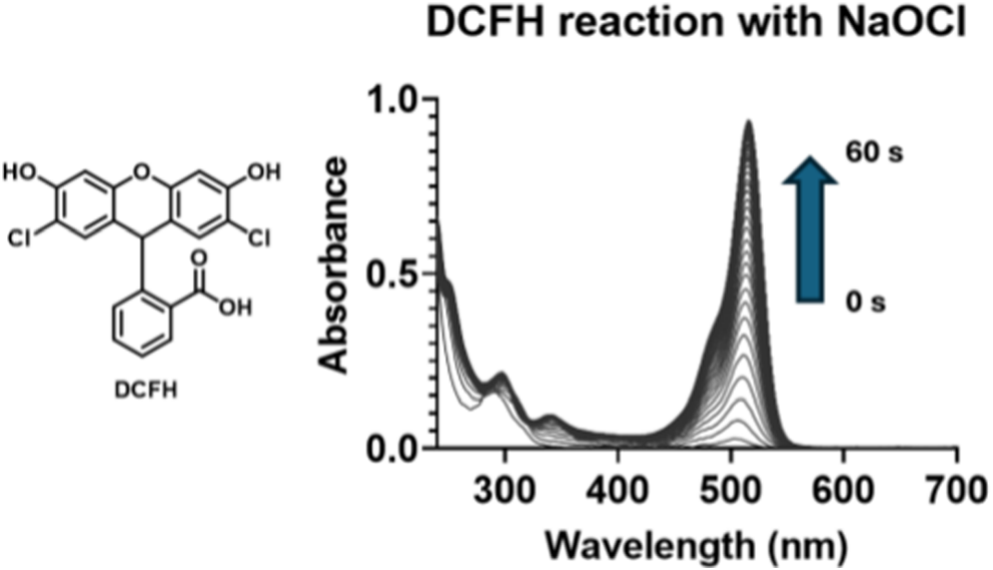
DCFH reaction with NaOCl monitored by using stopped flow in PBS at pH 7.4 and 37 °C and the determined second-order rate constant is 580 ± 18 M^–1^ s^–1^.

### Buffer Effect on DCFH Oxidation with NaOCl

We studied the effect of buffer reactivity on DCFH oxidation by incubating 10 μM DCFH with 100 μM NaOCl in different buffer solutions at 37 °C. Ninety-six-well plates were used for this study. We first used an experimental protocol designed to examine the effect of the buffer without having the competitive reaction of DCFH with NaOCl. Specifically, 50 μL of 400 μM NaOCl was first incubated with 100 μL of a 10 mM buffer at 37 °C for 5 min. Subsequently, 50 μL of 40 μM DCFH was added to the solution before recording of the fluorescence using a plate reader (λ_ex_ 495 nm and λ_em_ 530 nm). As expected, NaOCl led to no fluorescent turn-on effect of the probe when the experiments were conducted in HEPES, MES, Tris-Cl, or ammonium acetate buffer, indicating consumption of NaOCl by the buffer molecules (Figure 8) and the inability of the chloramine products to react with DCFH, whereas the same experiments in PBS and citrate buffer showed fluorescence turn on (Figure 8). Next, a similar experiment was conducted without the incubation step of the buffer and ROS (Figure S14). Briefly, 50 μL of 400 μM NaOCl was added first to 100 μL of 10 mM buffer. This was immediately followed by the addition of 50 μL of 40 μM DCFH and then recording of the fluorescence using a plate reader (λ_ex_ = 495 nm and λ_em_ = 530 nm). The results were such that the highest fluorescence signal was observed in PBS followed by citrate buffer (Figure 8). In MES buffer, the fluorescence intensity was about 50% of that in PBS. In other buffers, including HEPES, Tris-Cl, and ammonium acetate, no significant fluorescence turn-on of the probe by NaOCl was observed, indicating consumption of NaOCl by the buffer molecules (Figure 8). These results are in-line with the reaction kinetics as MES showed a slower reaction rate with NaOCl compared to HEPES, Tris-Cl, and ammonium acetate (Figure 3 and Table 1). Furthermore, the results indicate that these buffer molecules can cause interference of variable degrees depending on the experimental protocols. Overall, the results demonstrate strong interference from four buffers: HEPES, MES, Tris-Cl, and ammonium acetate.

**Figure 8:**
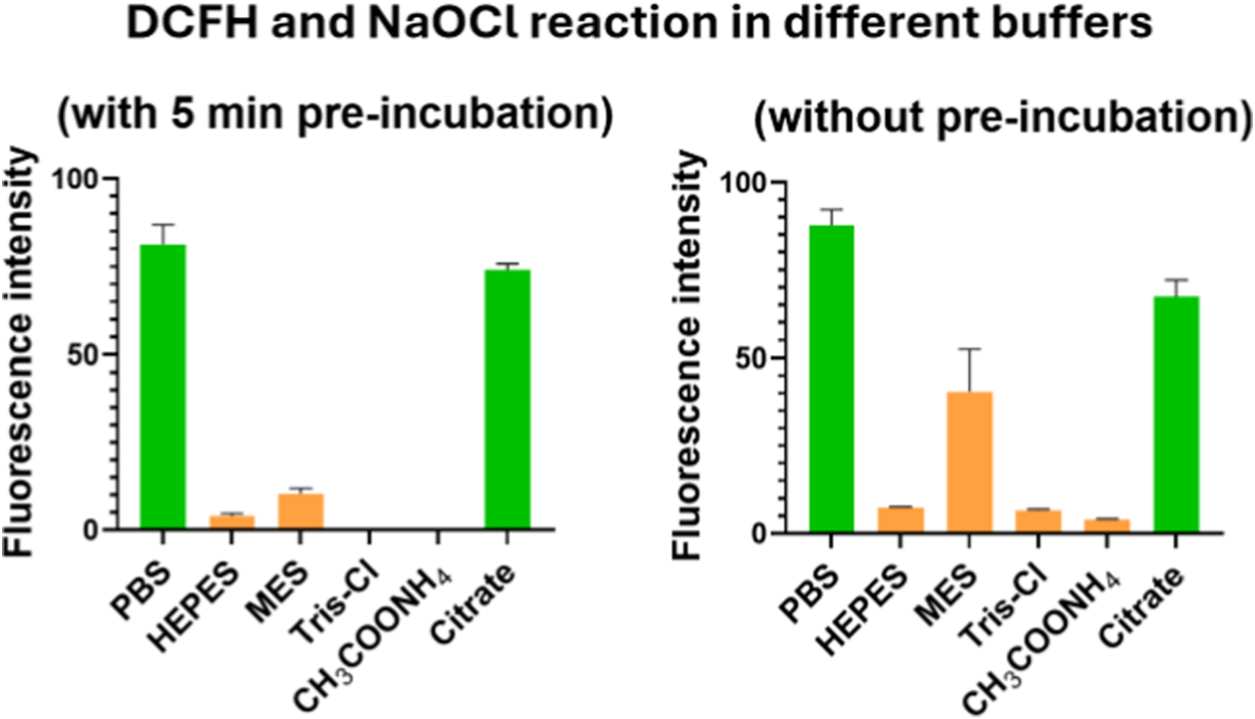
Effect of the Buffer Reactivity on DCFH oxidation by NaOCl.

## Conclusions

ROS research relies on using reaction-based probes for determining its concentrations. However, the exact meaning of the concentrations derived from different methods may vary depending on specific probes used and the specific experimental conditions.^27^ As such, direct comparisons of ROS concentration values across multiple studies are not always feasible because of the need to carefully control all factors that could lead to variations in the final outcome. In an effort to identify factors that could lead to skewed results, we have recently described how an organic solvent such as DMSO could impact experimental outcomes.^29^ Work conducted in this study using the two most abundant ROS (H_2_O_2_ and NaOCl) and ONOO^–^ in several commonly used buffer solutions demonstrates that HEPES, Tris, MES, and ammonium acetate can cause severe interference problems. These buffer solutions can lead to false negative results when tested for ONOO^–^ and the second most abundant ROS: NaOCl. Furthermore, these buffer components interact with boronate in its reaction with H_2_O_2,_ leading to significant interference problems. We should also note that such possible interference problems may extend to ROS concentration determination in cell or tissue lysates. For example, HEPES and Tris are commonly used buffering agents in formulations for cell culture work. For example, 50 mM Tris-Cl is used in RIPA lysis buffer^81,82^ and 25 mM HEPES is used in a few of DMEM formulations.^83,84^ One would expect interference by Tris-Cl or HEPES in ROS detection studies in cell lysates prepared by using such formulations.

We should note that our study is not meant to be comprehensive in providing guidelines for future work. Instead, it is meant to highlight the need to pay attention to the buffer used because of their chemical reactivity with certain ROS. We hope that the results will help others design their own experiments in ROS research in an effort to minimize potential problems. We also hope to stimulate additional studies to bring improved rigor to working with ROS.

## Supplementary Material



## Notes

Reaction rate determination studies; experimental protocols; general information, including materials, instruments, and methods (PDF)
