# Supplementary material for: Detection and Analysis of Reactive Oxygen Species (ROS): Buffer Components Are Not Bystanders
Source: Anal Chem. 2025 Jun 3;97(28):14931–42. doi: 10.1021/acs.analchem.4c07070 (PMC12291042; doi:10.1021/acs.analchem.4c07070)
Supplement: Supplementary file 1 [file ac4c07070_si_001.pdf]

Supporting information

Detection and Analysis of Reactive Oxygen Species (ROS): Buffer Components Are Not Bystanders

Shubham Bansal, Muskan Gori, Joanna Afokai Quaye, Giovanni Gadda, and Binghe Wang*

Department of Chemistry and Center for Diagnostics and Therapeutics, Georgia State University, Atlanta, Georgia 30301 USA

*Address correspondence to
Dr. Binghe Wang
Regents Professor and Dr. Frank Hannah Chair
Georgia Research Alliance Eminent Scholar
Department of Chemistry
Georgia State University
Atlanta, Georgia 30301
USA
wang@gsu.edu
Phone: 404-413-5544
ORCID: 0000-0002-2200-5270

Table of Contents

1. Experimental Section 2
1.1. General information of materials, instruments, and methods 2
1.2. Buffer solutions 3
1.3. Studies of buffer reactivity with NaOCl using UV-Vis spectrophotometry 4
1.4. Studies of ACN reactivity with NaOCl using UV-Vis spectrophotometry 4
1.5. NaOCl reaction kinetics with commonly used buffer components using stopped-flow 4
1.6. Second order rate constant of the MES or HEPES reaction with NaOCl 5
1.7. Boronate reaction with HOCl in 0.1% TFA or 0.1 M HCl 5
1.8. DCFH oxidation kinetics with NaOCl 5
1.9. Buffer effect on DCFH oxidation by NaOCl 6
1.10. Boronate reaction with $ONOO^-$ in commonly used buffer solutions 6
2. NaOCl reaction kinetics with commonly used buffer compounds 7
3. Rate constant of HEPES reaction with NaOCl 7
4. Second-order rate constant of HEPES reaction with NaOCl 8
4.1. Pseudo first order rate constant of NaOCl reaction with HEPES at varying concentrations 8
4.2. Second-order rate constant of NaOCl reaction with HEPES 8
5. Rate constant of MES reaction with NaOCl 9
6. Second-order rate constant of MES reaction with NaOCl 9
6.1. Pseudo first order rate constant of NaOCl reaction with MES at varying concentrations 9

6.2. Second-order rate constant of NaOCl reaction with MES 10
7. Stability of NaOCl with ACN 10
8. Effect of 0.1% TFA on NaOCl reaction with boronate 11
9. Boronate reaction with NaOCl in citrate buffer 11
10. Boronate reaction with NaOCl in PBS 12
11. Rate constant of DCFH reaction with NaOCl in PBS 12
11.1. Pseudo first order rate constant of DCFH reaction with NaOCl at varying concentrations 12
11.2. Second-order rate constant of DCFH reaction with NaOCl 13
12. DCFH reaction with NaOCl in different buffers 13
13. References 13

1. Experimental Section

1.1. General information of materials, instruments, and methods

Material: All reagents and solvents were of reagent grade from Sigma-Aldrich (Massachusetts, USA) or Oakwood Products, Inc. (South Carolina, USA). 4-acetylphenylboronic pinacolate ester (APBE) **1** was purchased from Ambeed (Illinois, USA). 2,7-Dichlorodihydrofluorescein (DCFH, Synonym with DCFH2, CAS 106070-31-9, and Catalog No. HY-153006) was purchased from MedChemExpress (New Jersey, USA). NaOCl solution (CAS 7681-52-9) was purchased from Sigma-Aldrich (Massachusetts, USA). The concentration of NaOCl solution was determined to be 1.811 M using UV-Vis spectrophotometer at 292 nm (ε: 360 $M^{-1}cm^{-1}$). ONOO- was prepared and quantified to be 122 mM using UV-Vis spectrophotometer at 302 nm (ε: 1670 $M^{-1}cm^{-1}$) by following the reported procedure.[1]

Instruments: Samples of milligram-quantities were weighed on C-33 microbalance (CAHN instruments Inc., California, USA). For pH measurement, Orion Star A111 pH meter equipped with ORION 8157BNUMD ROSS Ultra pH/ATC Triode was used (Thermo Scientific, Massachusetts, USA). For stopped-flow experiments, thermostated SF-61DX2 Hi-Tech KinetAsyst high-performance stopped-flow spectrophotometer equipped with a photomultiplier detector was used. Plate reader measurements were performed using VARIOSKAN LUX from Thermo Scientific (Massachusetts, USA). For UV-Vis measurements were performed on Shimadzu UV-1900i UV-Vis spectrophotometer (Kyoto, Japan). HPLC analysis was performed using Shimadzu LC-2050C 3D liquid chromatograph system (Kyoto, Japan). Column: Sunfire C18 3.5 μm, 3.0 × 150 mm.

Methods: The HPLC mobile phase was made of A: H_2O (0.1% TFA) and B: ACN (0.1% TFA). Flow rate 0.5 mL/min. Gradient methods used: (Method A) 22% B, 0-1 min; 28% B, 1-9 min; 95% B, 9.0-9.1 min; 95% B, 9.1-12.0 min; 5% B, 12.0-12.1 min; 5% B, 12.1-15 min. (Method B) 95% B, 0-10 min; 95% B, 10-12 min; 5% B, 12-12.1 min; 5% B, 12.1-15 min. Buffers were used at the following pH: HEPES at 7.4; Tris-Cl at 7.4; PBS at 7.4; MES at 6.3; citrate buffer at 5.9; and ammonium acetate buffer at 4.5. For DCFH fluorescence measurements, excitation wavelength 495 nm and emission wavelength 530 nm were used.

1.2. Buffer solutions

The following protocol was used to prepare buffer solutions.

HEPES buffer, pH 7.4: 1 M HEPES buffer at pH 7.4 was purchased from Sigma-Aldrich (Massachusetts, USA). Subsequent dilutions were prepared according to the individual experiment.

PBS buffer, pH 7.4: 100 mM and 10 mM were purchased from Corning (New York, USA). Subsequent dilutions were prepared according to the individual experiment.

Tris-Cl buffer, pH 7.4: Firstly, 1 M Tris buffer was prepared by dissolving 4 g of Sodium chloride, 0.1 g of Potassium Chloride, and 1.5 g of Tris base in 400 ml water. Then the pH was adjusted using conc. HCl to pH 7.4 followed by the addition of water till the solution had 500 mL total volume. Subsequent dilutions were prepared according to the individual experiment.

MES, pH 6.3: Firstly, 1 M MES buffer was prepared by dissolving 4.26 g of MES monohydrate in 15 ml water. Then the pH was adjusted using 10 N NaOH followed by the addition of water till the solution had a 20 mL total volume. Subsequent dilutions were prepared according to the individual experiment.

Citrate buffer, pH 5.9: Firstly, 1 M Citrate buffer was prepared by dissolving 63.88 g of tri sodium citrate dihydrate and 6.30 g of citric acid in 200 mL of water. Then the pH was adjusted using 10 N NaOH to pH 5.9 followed by the addition of water till the solution had 250 mL of total volume. Subsequent dilutions were prepared according to the individual experiment.

Ammonium acetate buffer, pH 4.5: Firstly, 0.1 M of ammonium acetate was prepared by dissolving 2.886 g of ammonium acetate and 0.889 g of acetic acid were dissolved in 400 mL of

water. Then the pH was adjusted to 4.5 by using 1 N HCl followed by water till the solution had 500 mL total volume. Subsequent dilutions were prepared according to the individual experiment.

1.3. Studies of buffer reactivity with NaOCl using UV-Vis spectrophotometry

Firstly, stock solutions were prepared in H_2O. 100 mM of NaOCl was prepared by adding 55 μL of NaOCl (from 1.811 M) to 945 μL of H_2O. 1 M stock solutions of HEPES, MES, Tris-Cl and citrate were prepared in H_2O, respectively. 100 mM stock solution was prepared for ammonium acetate and PBS.

The following protocol was used for the reaction of NaOCl with HEPES/MES/Tris-Cl/Citrate buffer, respectively.

For preparing a mixture of 2 mM NaOCl and 20 mM buffer, 60 μL of NaOCl stock solution (100 mM) was added to 2.880 mL of H_2O, followed by the addition of 60 μL of the buffer stock solution (1 M). Then the mixture was incubated at 37 °C. UV spectra were recorded at pre-designated time points.

The following protocol was used for the reaction of NaOCl with PBS or ammonium acetate buffer, respectively.

For preparing a mixture of 2 mM NaOCl and 20 mM buffer, 60 μL of NaOCl stock solution (100 mM) was added to 2.340 mL of H_2O followed by the addition of 600 μL of a buffer stock solution (0.1 M). Then the reaction mixture was incubated at 37 °C. UV spectra were recorded at pre-designated time points.

1.4. Studies of ACN reactivity with NaOCl using UV-Vis spectrophotometry

Firstly, stock solutions were prepared in H_2O. 100 mM of NaOCl was prepared by adding 55 μL of NaOCl (from 1.811 M) to 945 μL of H_2O. For preparing a mixture of 2 mM NaOCl in 20% ACN in PBS, 60 μL of NaOCl stock solution (100 mM) was added to 2.940 mL of PBS:ACN (8:2) at pH 7.4. Then the mixture was incubated at 37 °C. UV spectra were recorded at pre-designated time points.

1.5. NaOCl reaction kinetics with commonly used buffer components using stopped-flow

Firstly, working solutions were prepared in H_2O at 2× the final reaction solution. 2 mM of NaOCl was prepared by adding 55 μL of NaOCl stock solution (1.811 M) to 49.945 mL of H_2O.

40 mM solutions were prepared in H_2O for the following buffer components: HEPES, MES, Tris-Cl and citrate. Solutions were loaded into syringes and mounted into the stopped-flow spectrophotometer. Then the reaction was followed by monitoring the decay of the NaOCl peak at 325 nm. For the data analysis, time resolved NaOCl decay was fitted into one-phase exponential Equation 1 (for HEPES) and two-phase exponential Equation 2 (for MES). Where k represents the reaction rates; A represents the absorbance change; and C represents the final absorbance.

Equation 1, for one-phase exponential decay: $-A*\exp(-k*X) + C$

Equation 2, for two-phase exponential decay: $-A_1*\exp(-k_1*X) + -A_2*\exp(-k_2*X) + C$

1.6. Second order rate constant of the MES or HEPES reaction with NaOCl

Firstly, working solutions were prepared in H_2O at 2× the final reaction concentrations. 2 mM of NaOCl was prepared by adding 55 µL of NaOCl stock solution (1.811 M) to 49.945 mL of H_2O. MES or HEPES was prepared at the following concentrations: 50 mM, 60 mM, 70 mM, 80 mM, 90 mM, 100 mM, and 120 mM. Solutions were loaded into syringes and mounted into the stopped-flow spectrophotometer. Then reaction was followed by monitoring the decay of the NaOCl peak at 325 nm. For the data analysis, time resolved NaOCl decay was fitted into one-phase exponential decay Equation 1 (for HEPES) and two-phase exponential Equation 2 (for MES).

1.7. Boronate reaction with HOCl in 0.1% TFA or 0.1 M HCl

Firstly, stock solutions were prepared. 10-mM solution of APBE **1** was prepared in DMF and 10-mM NaOCl was prepared in H_2O. Then, 10 µL was taken from the 10-mM stock solution of NaOCl added to 980 µL of 0.1% TFA or 0.1 M HCl in H_2O. This was followed by the addition of 10 µL of 10-mM APBE **1** solution. The reaction mixture had a final concentration of 100 µM of APBE **1** and 100 µM of HOCl in 0.1% TFA or 0.1 M HCl containing 1% DMF at 37 °C. Then, 20 µL aliquot was drawn from the reaction mixture and injected into HPLC. Mobile phase gradient method A was used to monitor the product formation.

1.8. DCFH oxidation kinetics with NaOCl

Firstly, stock solutions were prepared. 10-mM stock solution of DCFH was prepared in DMF by dissolving 1.083 mg in 268 µL of DMF. 40-µM working solution of DCFH was prepared in PBS by adding 40 µL of DCFH stock solution (10 mM) to 9.960 mL of PBS. 10-mM stock solution of NaOCl was prepared by adding 55 µL of NaOCl stock solution (1.811 M) to 9.945 mL of PBS.

Working solutions of NaOCl were prepared in PBS at the following concentrations: 500 μM, 600 μM, 700 μM, 800 μM, 900 μM, and 1 mM. Solutions were loaded into syringes and mounted into the stopped-flow spectrophotometer. Then reaction was then followed by the formation of new peak at 515 nm. For the data analysis, time resolved peak formation at 515 nm was fitted into Equation 1.

1.9. Buffer effect on DCFH oxidation by NaOCl

Firstly, stock solutions were prepared. 10-mM stock solution of DCFH was prepared in DMF by dissolving 1.083 mg in 268 μL of DMF. 40-μM working solution of DCFH was prepared in H_2O by adding 20 μL of DCFH stock solution (10 mM) to 4.980 mL of H_2O. For NaOCl, firstly a 100-mM stock solution of NaOCl was prepared by adding 55 μL of NaOCl (1.811 M) to 945 μL of H_2O. Then a 10-mM NaOCl solution was prepared by adding 100 μL of the NaOCl stock solution (100 mM) to 900 μL of H_2O. A working solution of NaOCl was prepared at 400 μM by adding 280 μL of the NaOCl stock solution (10 mM) to 6.72 ml of H_2O. The experiments were carried out using a 96-well plate. Firstly, 100 μL of 10 mM buffer was added to a well. Then to the same well 50 μL of the NaOCl working solution (400 μM) was added. The plate was then incubated for 5 min at 37 °C. This was followed by the addition of 50 μL of DCFH stock solution (40 μM). The reaction mixture had a final concentration of 10 μM of DCFH and 100 μM of NaOCl in 5 mM buffer with 0.1% DMF. Fluorescence measurements were conducted using a plate reader (λ_{ex} 495 nm and λ_{em} 530 nm). Similar procedures were followed for the experiments with each buffer component. All the buffer solutions were at 5 mM final concentration. A similar experiment was carried out without the buffer and ROS pre-incubation step. The experiments were carried out in triplicates and in each trial, reactions were carried out in duplicates.

1.10. Boronate reaction with $ONOO^-$ in commonly used buffer solutions

Firstly, stock solutions were prepared. 10-mM solution of APBE **1** was prepared in DMF. 10 mM working solution of $ONOO^-$ was prepared in H_2O by adding 82 μL of $ONOO^-$ (122 mM) in 918 μL of H_2O. Then, 10 μL was taken from the 10-mM working solution of $ONOO^-$ and added to 980 μL of buffer. This was followed by the addition of 10 μL of 10 mM APBE **1** solution. The reaction mixture had a final concentration of 100 μM of APBE **1** and 100 μM of $ONOO^-$ in 10 mM different buffer solutions containing 1% DMF at 37 °C. Then, 20 μL aliquot was drawn from

the reaction mixture and injected into HPLC. Mobile phase gradient method A was used to monitor the product formation.

2. NaOCl reaction kinetics with commonly used buffer compounds

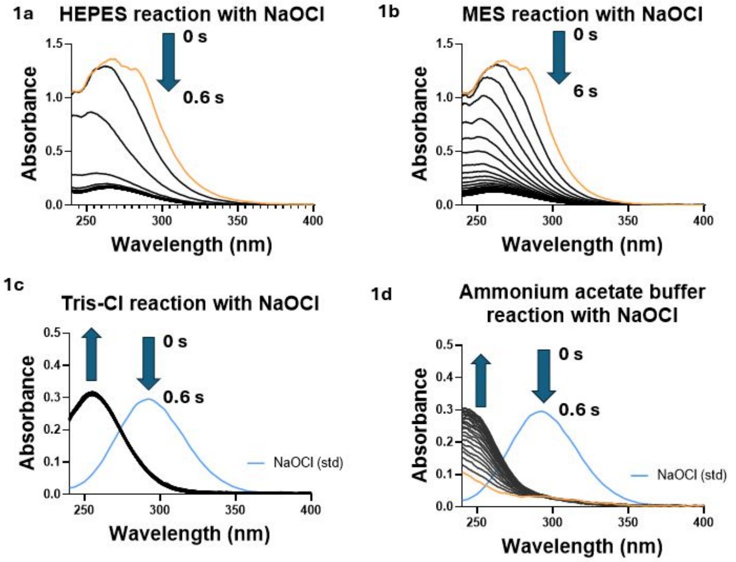

Figure S1. Reaction of buffer compounds with NaOCl monitored by stopped flow. (1a) HEPES (20 mM) reaction with NaOCl (1 mM) at pH 7.4, 37 °C. (1b) MES (20 mM) reaction with NaOCl (1 mM) at pH 6.3, 37 °C. (1c) Tris-Cl (20 mM) reaction with NaOCl (1 mM) at pH 7.4, 37 °C. (1d) Ammonium acetate (20 mM) reaction with NaOCl (1 mM) at pH 4.5, 37 °C.

3. Rate constant of HEPES reaction with NaOCl

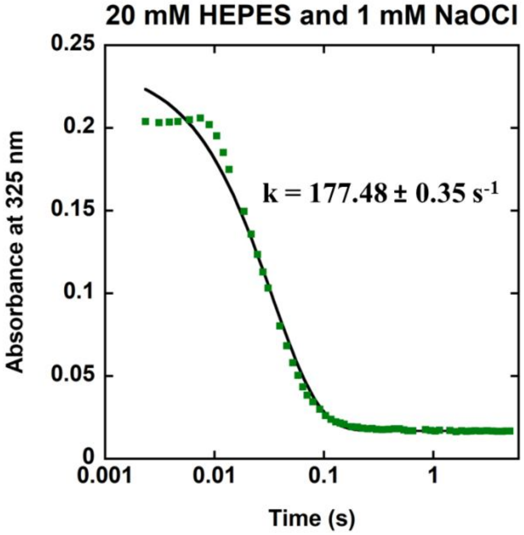

Figure S2. Rate constant of HEPES reaction with NaOCl determined by using stopped flow spectrometry. Rate was determined for 20 mM HEPES reaction with 1 mM NaOCl at pH 7.4, 37 °C. Data are presented as an average of 6 runs. The x-axis is presented in log scale. Rate constant was determined using a single exponential equation (Equation 1, described in the experimental section).

4. Second-order rate constant of HEPES reaction with NaOCl

4.1. Pseudo first order rate constant of NaOCl reaction with HEPES at varying concentrations

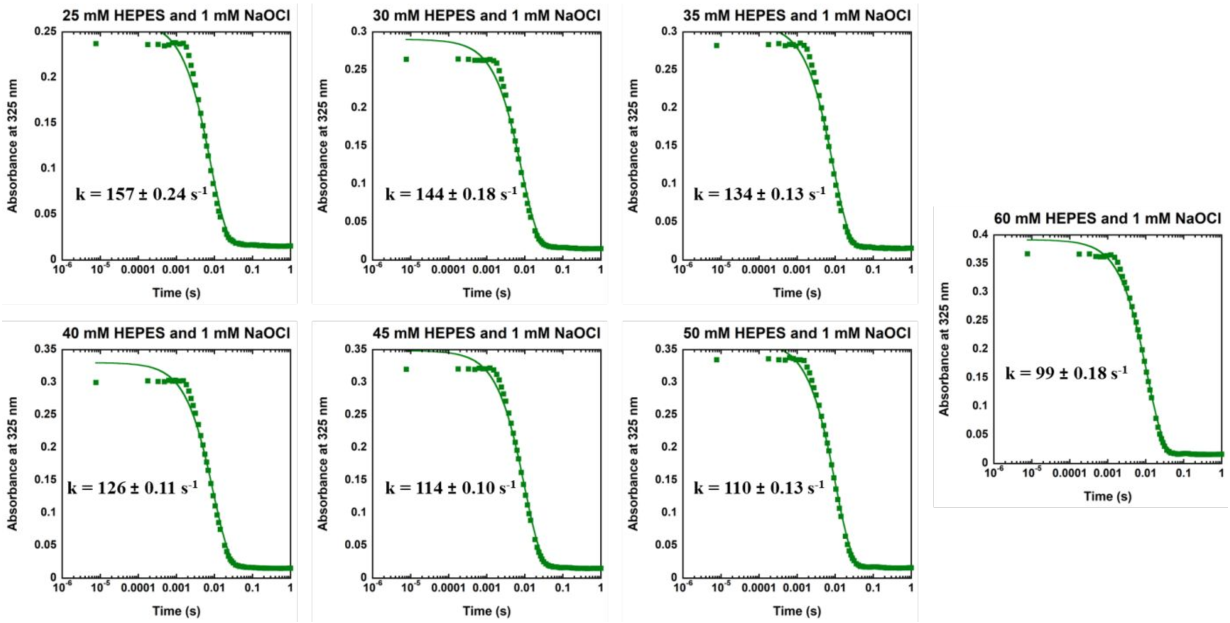

Figure S3. Rate constant determination of 1 mM NaOCl reaction with HEPES at 25 mM, 30 mM, 35 mM, 40 mM, 45 mM, 50 mM, and 60 mM and pH 7.4 (37 °C). Each data set is presented as an average of 6 runs. The x-axis is presented in log scale. The rate constant was determined using a single exponential equation (Equation 1, described in the experimental section).

4.2. Second-order rate constant of NaOCl reaction with HEPES

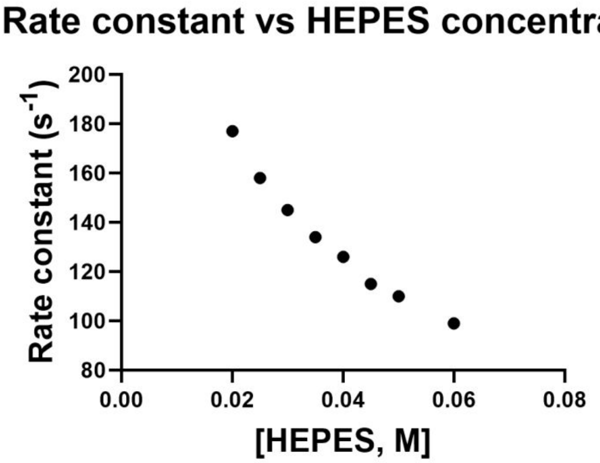

Figure S4. Second order rate constant determination of HEPES reaction with NaOCl at pH 7.4, 37 °C. Detailed conditions are described in Figure S3.

5. Rate constant of MES reaction with NaOCl

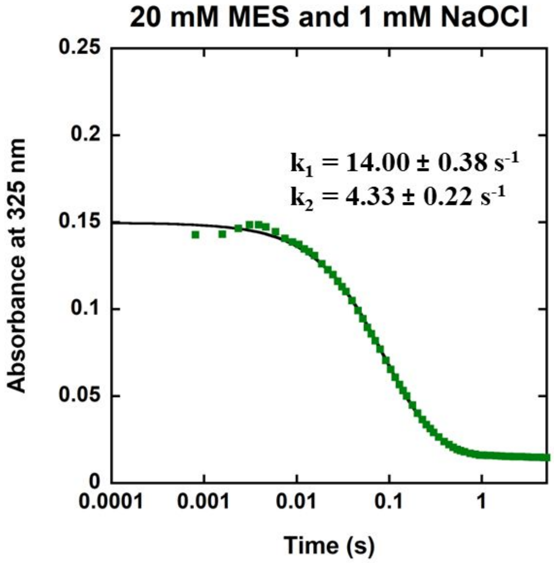

Figure S5. Rate constant of MES reaction with NaOCl determined by using stopped flow spectrometry. Rate was determined for 20 mM MES reaction with 1 mM NaOCl at pH 6.3, 37 °C. Data are presented as an average of 6 runs. The x-axis is presented in log scale. Rate constant was determined using double exponential equation (Equation 2, described in the experimental section).

6. Second-order rate constant of MES reaction with NaOCl

6.1. Pseudo first order rate constant of NaOCl reaction with MES at varying concentrations

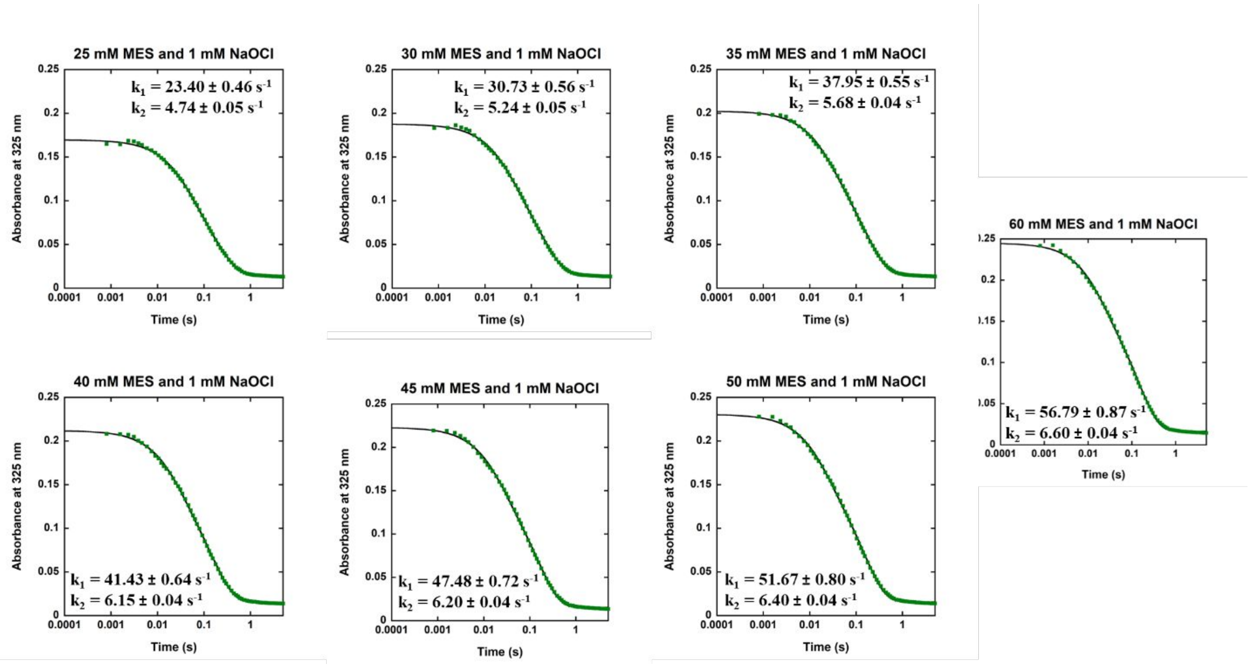

Figure S6. Rate constant determination of 1 mM NaOCl reaction with MES at 25 mM, 30 mM, 35 mM, 40 mM, 45 mM, 50 mM, and 60 mM and at pH 6.3 (37 °C). Each data set is presented as an average of 6 runs. The x-axis is presented

in log scale. Rate constant was determined using double exponential equation (Equation 2, described in the experimental section).

6.2. Second-order rate constant of NaOCl reaction with MES

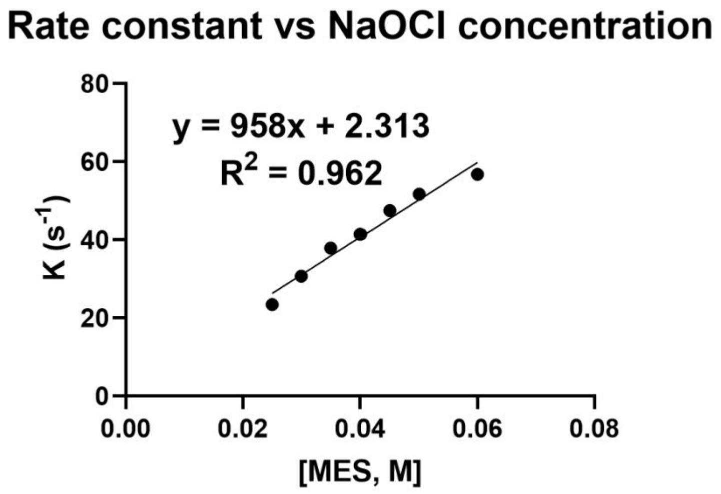

Figure S7. Second-order rate constant determination of MES reaction with NaOCl at pH 6.3, 37 °C. Detailed conditions are described in Figure S6.

7. Stability of NaOCl with ACN

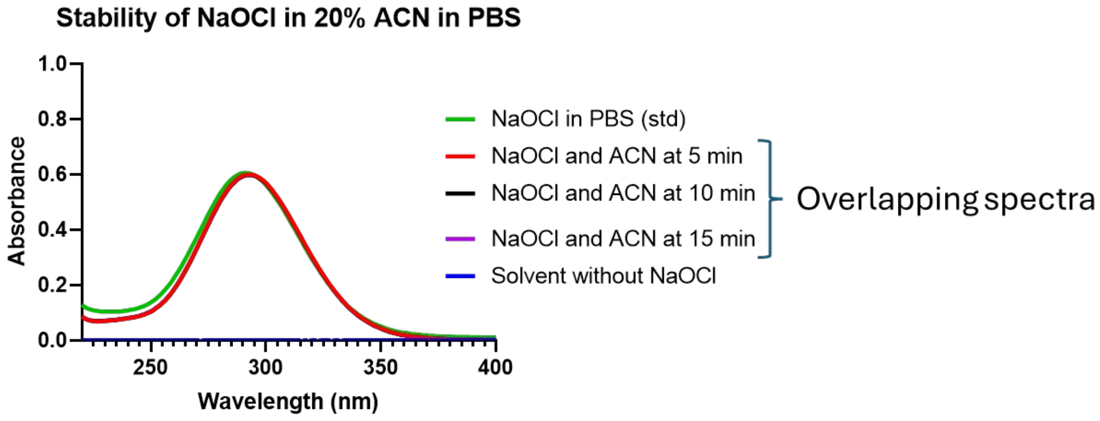

Figure S8. Stability of NaOCl in 20% ACN in PBS at pH 7.4, 37 °C.

8. Effect of 0.1% TFA on NaOCl reaction with boronate

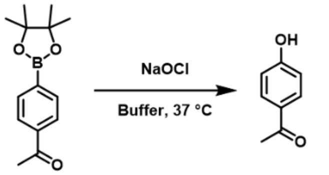

1 2

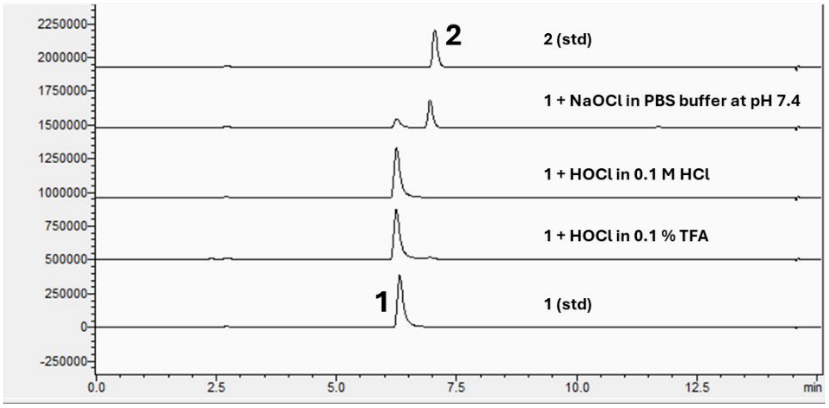

Figure S9. HLPC chromatograms showing the effect of 0.1% TFA on boronate reaction with NaOCl. The reaction of NaOCl with carboxylic acids has been reported to be slow with the second order rate constant of 3.3×10^{-3} $M^{-1}s^{-1}$ for the reaction of formic acid with NaOCl.[2] This reaction kinetic indicates that TFA should not directly consume the NaOCl. Nevertheless, NaOCl under acidic conditions exists in protonated form (HOCl), which will be several folds less nucleophilic compared to NaOCl for boronate. Overall, the results indicate that TFA can act as reaction quencher as the boronate oxidation was not observed in the experimental time frame.

9. Boronate reaction with NaOCl in citrate buffer

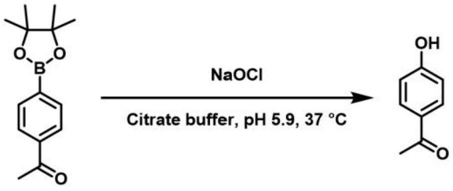

1 2

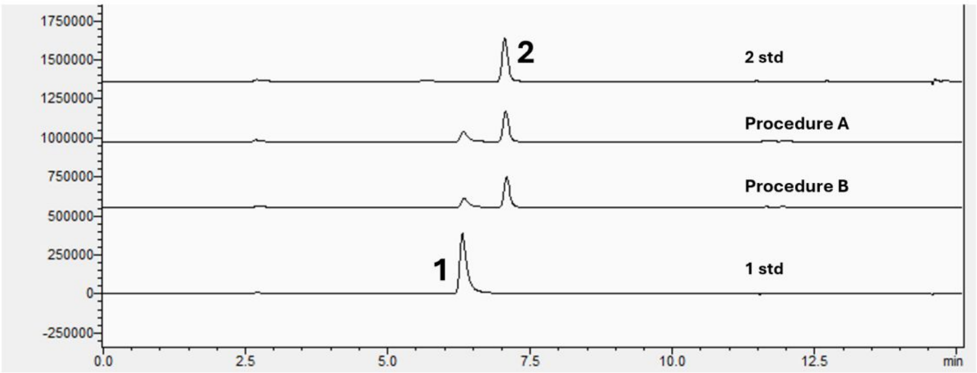

*Figure S10. HLPC chromatogram showing the effect of the order of reagent addition on boronate reaction with NaOCl in citrate buffer at pH 6.9, 37 °C. Experimental procedure A: NaOCl was incubated with citrate buffer for 15 min at 37 °C before the addition of compound **1**. Experimental procedure B: Compound **1** was added to the citrate buffer before the addition of NaOCl.*

10. Boronate reaction with NaOCl in PBS

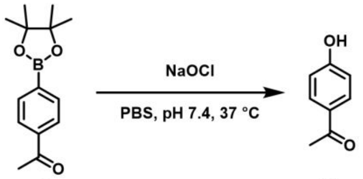

1 2

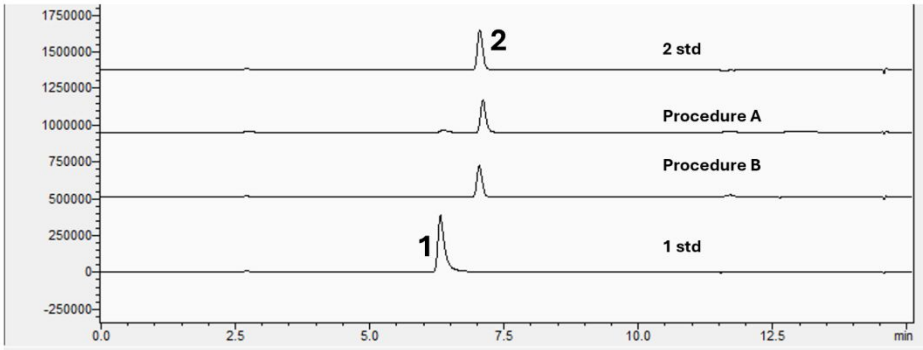

*Figure S11. HLPC chromatogram showing the effect of the order of reagent addition on boronate reaction with NaOCl in PBS at pH 7.4, 37 °C. Experimental procedure A: NaOCl was incubated with PBS for 15 min at 37 °C before the addition of compound **1.** Experimental procedure B: Compound **1** was added to PBS before the addition of NaOCl.*

11. Rate constant of DCFH reaction with NaOCl in PBS

11.1. Pseudo first order rate constant of DCFH reaction with NaOCl at varying concentrations

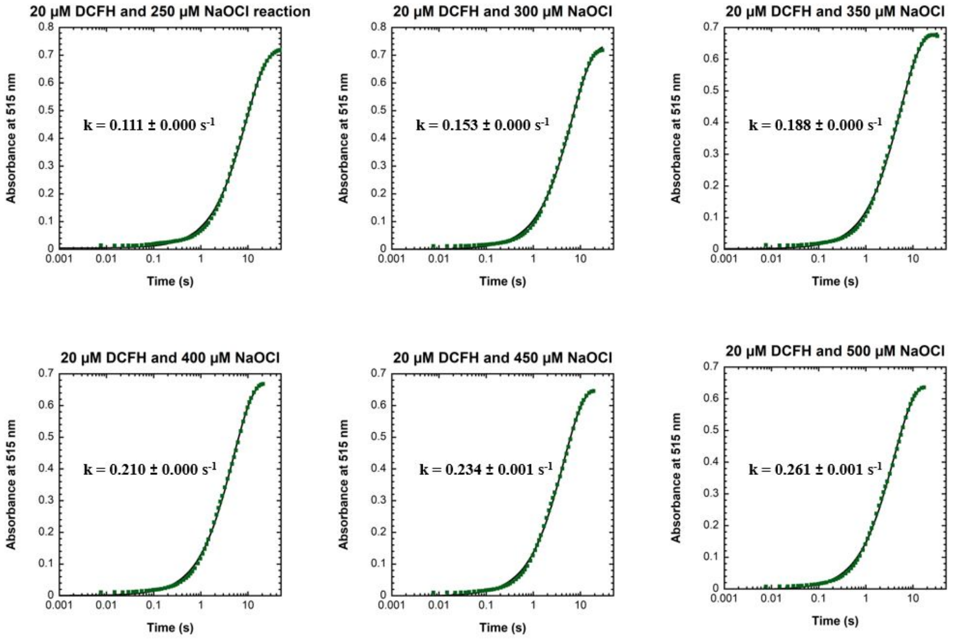

Figure S12. The rate constant of DCFH reaction with NaOCl (250 – 500 μM) in PBS at pH 7.4, 37 °C. Each data set is presented as an average of 3 runs. The x-axis is presented in a log scale. The rate constant was determined using a single exponential equation (Equation 1, described in the experimental section).

11.2. Second-order rate constant of DCFH reaction with NaOCl

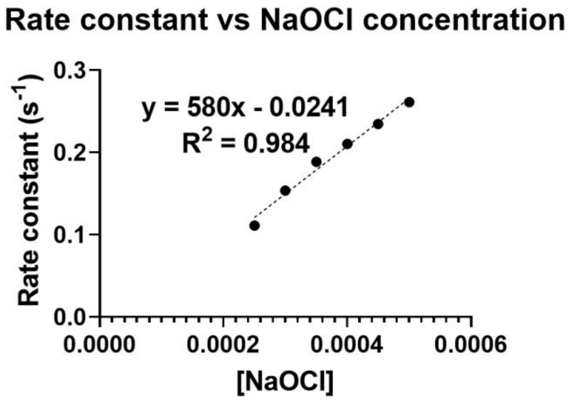

Figure S13. Second-order rate constant determination of DCFH reaction with NaOCl at pH 7.4, 37 °C. Detailed conditions are described in Figure S10.

12. DCFH reaction with NaOCl in different buffers

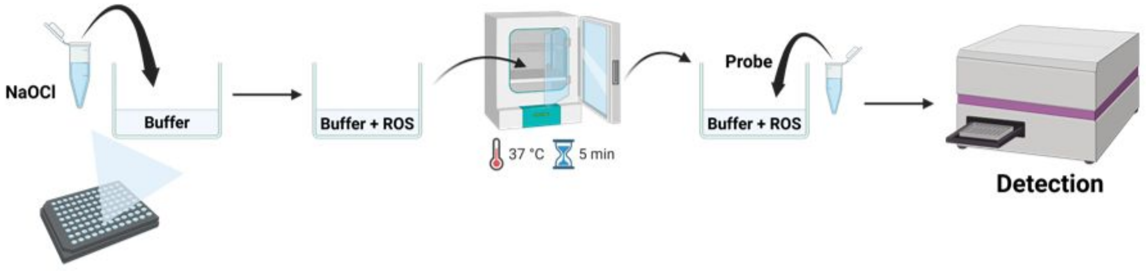

Figure S14. Experimental protocol for DCFH reaction with NaOCl in different buffers.

13. References

(1) Bruemmer, K. J.; Merrikhihaghi, S.; Lollar, C. T.; Morris, S. N.; Bauer, J. H.; Lippert, A. R. ^{19}F magnetic resonance probes for live-cell detection of peroxynitrite using an oxidative decarbonylation reaction. *Chem Commun (Camb)* **2014**, *50* (82), 12311-12314.
(2) Zhou, Z.; Jääskeläinen, A.-S.; Vuorinen, T. Oxidation of Cellulose and Carboxylic Acids by Hypochlorous Acid: Kinetics and Mechanisms. *Journal of Pulp and Paper Science* **2008**, *34*, 212-218.
